# Testing a relational account of search templates in visual foraging

**DOI:** 10.1038/s41598-023-38362-9

**Published:** 2023-08-02

**Authors:** Inga M. Grössle, Anna Schubö, Jan Tünnermann

**Affiliations:** Cognitive Neuroscience of Perception and Action, Department of Psychology, Philipps-University Marburg, Gutenbergstraße 18, 35032 Marburg, Germany

**Keywords:** Psychology, Human behaviour

## Abstract

Search templates guide human visual attention toward relevant targets. Templates are often seen as encoding exact target features, but recent studies suggest that templates rather contain “relational properties” (e.g., they facilitate “redder” stimuli instead of specific hues of red). Such relational guidance seems helpful in naturalistic searches where illumination or perspective renders exact feature values unreliable. So far relational guidance has only been demonstrated in rather artificial single-target search tasks with briefly flashed displays. Here, we investigate whether relational guidance also occurs when humans interact with the search environment for longer durations to collect multiple target elements. In a visual foraging task, participants searched for and collected multiple targets among distractors of different relationships to the target colour. Distractors whose colour differed from the environment in the same direction as the targets reduced foraging efficiency to the same amount as distractors whose colour matched the target colour. Distractors that differed by the same colour distance but in the opposite direction of the target colour did not reduce efficiency. These findings provide evidence that search templates encode relational target features in naturalistic search tasks and suggest that attention guidance based on relational features is a common mode in dynamic, real-world search environments.

## Introduction

Humans and other organisms are confronted with highly complex visual input from which they have to select information relevant in the current situation. Selective visual attention is the mechanism that enables efficient visual selection in complex search environments. Attention has traditionally been investigated in visual search tasks, in which observers search for a single target object often defined by a particular feature or a combination of features. A large body of literature has shown that search relies on “templates”, memory representations that contain a combination of the relevant target features^1–5^, and recent reviews and definitions can be found in Wolfe^6^ and Liesefeld et al.^7^. Templates were often assumed to encode specific target features, for instance a target’s colour or orientation, and when the actual target features deviate from the template, search efficiency is reduced^8^. More specific target templates also seem to better shield the system against interference from salient distractors^9,10^. From this point of view, it seems reasonable to assume that search is most efficient when the template can be tuned as precisely as possible to closely match the target features. In the following, we use the term “feature-specific account” to refer to this idea.

Recent research has revealed that the most specific template might not always be the most efficient one^11^. Experiments showed that templates that encode “relational properties” of the target in relation to the context (e.g., the target being “redder” than objects in the environment) can facilitate search most (e.g., Becker^12^; Hamblin-Frohman & Becker^13^). Becker and colleagues have dubbed this the “relational account” of target templates in visual search. For instance, in Martin and Becker’s^14^ visual search task, observers searched for one target defined by colour in the presence of similarly shaped non-targets and a differently shaped distractor and reported the target by identifying a letter presented inside the target shape. The non-targets established the colour-context, by rendering the target for example “bluer” than the environment and encoding this target–context relationship in a relational template. The colour of the distractor was systematically varied to create either a match in relational properties with the target or a mismatch. In one condition, both the target and the distractor were “bluer” (on a blue–green scale) than the surrounding non-targets, with the distractor being “the bluest” object, therefore agreeing even better than the target with a potential relational “bluer” target template. In another condition, the distractor was “greener” than both the non-targets and the target, rendering it opposite to a relational target template. As predicted by the relational account, search times were slowed down by a distractor that matched the relational properties of the target, whereas a distractor of similar feature distance—and hence the same relevance according to the feature-specific account—in the opposite direction had no effect on target search.

The stronger impact on search times of distractors that have matching relational properties shows that they interfere more with attention guidance, supporting the idea of relational templates. Martin and Becker^14^ also found evidence in eye-tracking and EEG data that showed that relationally matching distractors captured both overt and covert attention. Such relational guidance might help the visual system to compensate for effects of varying lighting, perspective, and distance, which render templates with exact feature values unreliable under natural viewing conditions^12^.

Similar results were reported for size, colour, luminance, and shape^12,15–17^. However, not all studies find evidence for uniquely relational guidance. For instance, Yu et al.^18^ found that the template is first tuned to relational properties to isolate target candidates, while the subsequent matching-decision is based on a more feature-specific template. In this matching-decision, the template is only somewhat tuned away from the distractor features to increase target–distractor distinctiveness. Yu et al.^19^ showed that the use of off-veridical templates is task-adaptive. In their experiments the feature shift of the template (driven by relationally matching colour distractors) was more pronounced when the feature context did not provide a distinct additional feature (a clearly different orientation) which observers could use to guide their search. Meeter and Olivers^17^ found that keeping the relational properties of a target and non-targets constant in consecutive trials led to shorter reaction times compared to when relational properties were changed. Reaction times where even shorter when the exact target and non-target features were repeated, indicating a feature-specific facilitation on top of relational guidance. These results show that search is neither completely feature-specific, nor strictly relational, but that different mechanisms are at work at different processing stages. Whether and to which extent relational guidance occurs in more natural, dynamic, and continuous tasks is even less clear. In such tasks, the exposure to and interaction with the stimulus material for prolonged periods of time could help observers to enhance the precision of their templates, rendering them highly feature-specific and making relational-guidance unnecessary.

In traditional search tasks observers search for a single target object in a sequence of discrete and randomly arranged search displays; observers respond with speeded presses of arbitrarily assigned buttons. Natural search environments, however, often contain more than one target, require planning and execution of directed actions, and they involve strategic decisions about whether to continue or abort search. Visual foraging tasks recently gained popularity as they mitigate several of the drawbacks of single target visual search (cf. Kristjánsson, Björnsson, et al.^20^; Kristjánsson, Ólafsdóttir, et al.^21^; Kristjánsson et al.^22^; Wolfe^6^). In visual foraging tasks, multiple elements have to be collected within “patches”. A patch is typically a display that contains multiple targets (often of different types) among other objects, creating a search environment that allows investigating visual search in rich environments and more continuous tasks. Often, participants are instructed to collect the targets as quickly as possible, and they have the option to leave a patch and move to the next one (a fresh, target-filled display) when they feel that search gets too inefficient as more and more targets are removed. This adds a strategic component to the task. Theories of optimal foraging predict that foragers leave patches when their current intake gets too low. More specifically, the marginal value theorem (MVT) predicts that foragers leave the environment when the rate of return (intake per time) drops below the average rate in the environment^23^. Hence, the foraging paradigm not only reveals search efficiency in different conditions, but also how individuals integrate their performance into strategic decisions (patch-leaving behaviour, e.g., number of targets left behind in a patch).

Whether relational guidance emerges in visual foraging has not been investigated yet. Under such complex conditions, the benefits of relational guidance might be masked by other processes involved, or it might simply be unnecessary as the prolonged behaviour within one environment could enable observers to form and refine feature-specific templates, increasing their efficiency (cf. Hamblin-Frohman et al.^24^). However, if relational guidance serves to improve performance under varying lighting, perspective, and distance^12^, it also seems plausible that relational guidance occurs more prominently in tasks like foraging that are close to real-world settings.

**Figure 1:**
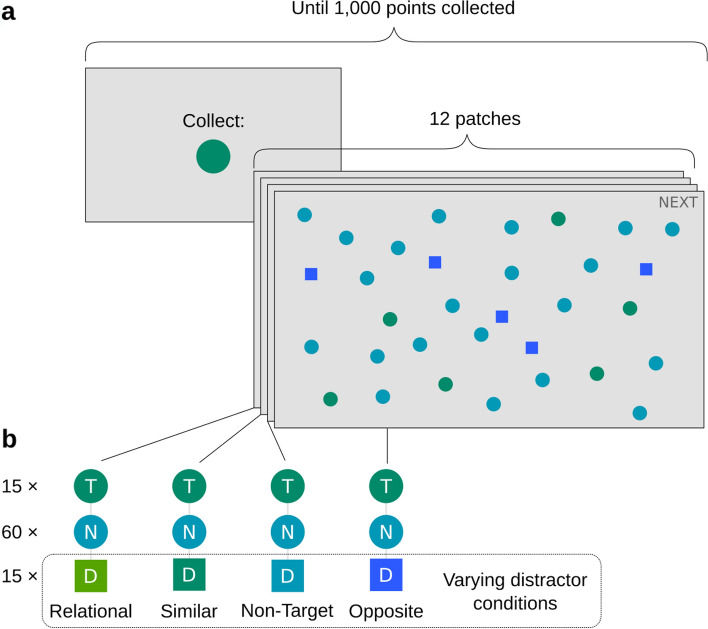
Stimuli and procedure: (**a**) Example trials. The first display in each block showed the target. Then participants searched through 12 patches. The figure shows the “Opposite” condition, with only a third of the items for illustration. Elements not to scale. (**b**) Colour combinations for all four conditions of olive-coloured targets. For aqua-coloured targets, non-target and distractor colours are accordingly switched. T = Target, N = non-target, D = distractor. Note: in the experiment stimuli were moving; colours are exaggerated for better differentiation. The same colour scheme and shapes as in this figure are used throughout the article for illustrations. The experiment also included trials with the opposite colour directions and reversed roles of circles and squares as targets and distractors. Figure best viewed in colour.

**Figure 2:**
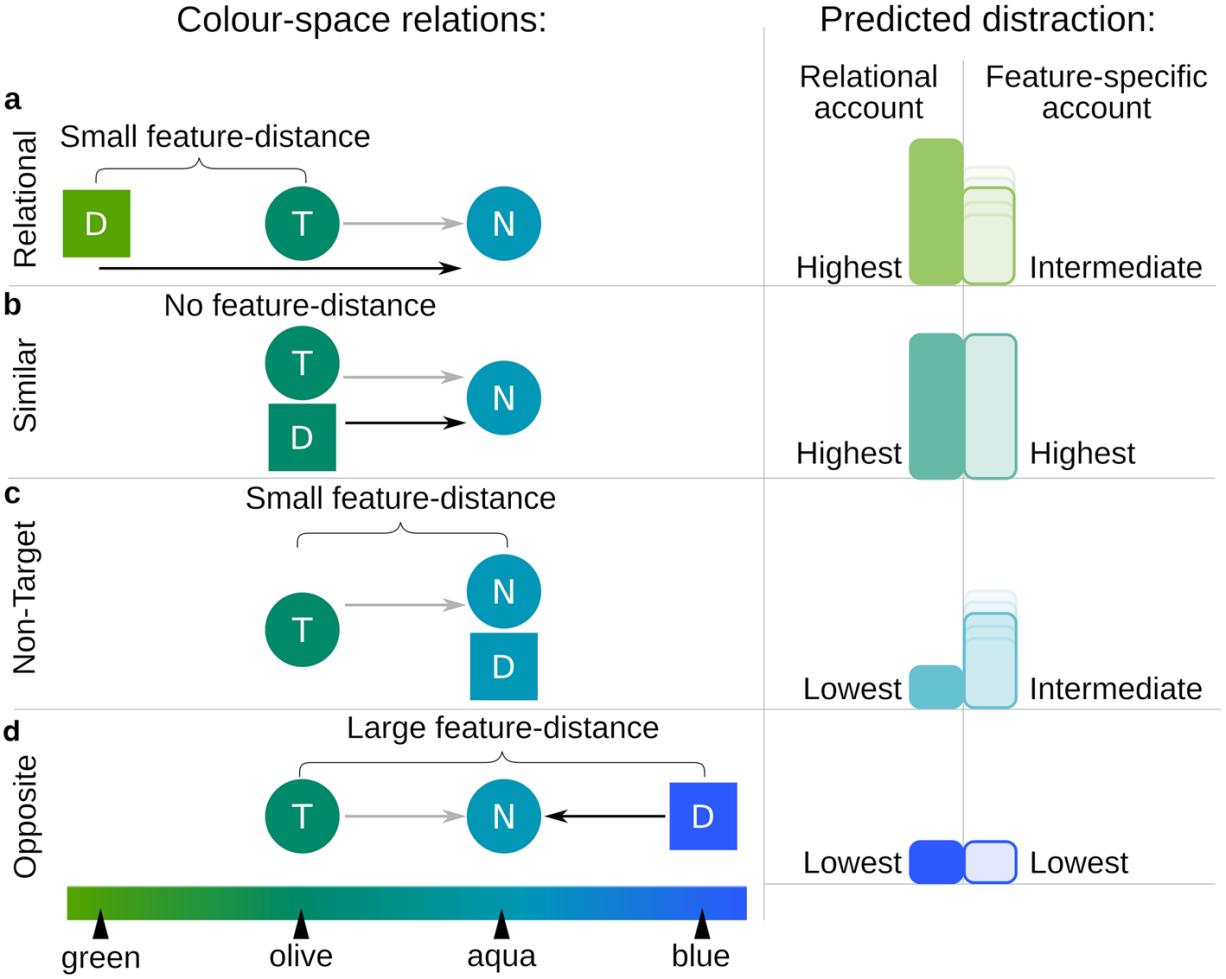
Colour-space relations and predicted pattern of distraction. Left panels: Grey arrows indicate the relation between targets and non-targets, which is the same in all conditions. Black arrows indicate relation between distractors and non-targets. (**a**) Relational condition: distractor colour differs from non-targets in the same direction as the target colour, feature-distance between target and distractor colour is small. (**b**) (Target-)Similar condition: distractors have the same colour as the targets, both differing from non-targets in the same direction. (**c**) Non-target condition: distractors have the same colour as non-targets. The feature-distance between target and distractor colour is small. (**d**) Opposite condition: distractor colour differs from non-targets in the opposite direction as the target colour, the feature-distance is large. T = Target, N = non-target, D = distractor. Right panels: The bar charts indicate the predicted distraction made by the relational and the feature-specific account. The bars visualize the expected order of differences (high, intermediate, low distraction) for conditions according to the two theoretical accounts. Intermediate distraction can be anywhere between the minimum and the maximum. This is indicated by the smearing of the vertical boundary of the intermediate bars.

Here we investigate whether target selection in foraging patches is guided in a relational or feature-specific manner by varying the target–distractor relationship. Foragers had to collect a total of 1000 points by clicking on the targets (one point per collected target) and were allowed to search through as many patches as they wanted and leave them at any time (see Fig. 1). Targets were specified by colour and shape, with non-targets differing in colour (but they had the same shape) to establish the relational context. Distractors differed in shape. Distractor colour was varied between patches (there was one distractor type per patch) to create four conditions with different feature distances and relationships between targets, distractors, and non-targets (see Fig. 2). The pattern of how well foragers performed in these conditions enables conclusions about the involvement of relational and feature-specific guidance (see Fig. 2). If guidance is purely feature-specific, then the amount of distractor interference would be inversely related to the feature-space distance to the target (indicated by the curly brackets in the left column of Fig. 2). If guidance is predominantly relational, distractor interference would depend only on whether the target and distractor differ in the same direction from the non-targets (see matching or non-matching directions of arrows in the left column of Fig. 2). In conditions where target and distractor differ in the same direction (Relational and Similar), distraction would be high, whereas no distraction is expected when they differ in opposite directions (Non-Target and Opposite).

As mentioned earlier, the foraging paradigm allows investigating patch leaving decisions and we want to explore how the relational properties in the patches affect them. In general, we expect foragers to leave patches, in agreement with the MVT, when the intake rate falls below the average intake rate of the environment. However, the different relational properties might inflict varying degrees of effort that could also influence patch leaving decisions.

## Results

**Figure 3:**
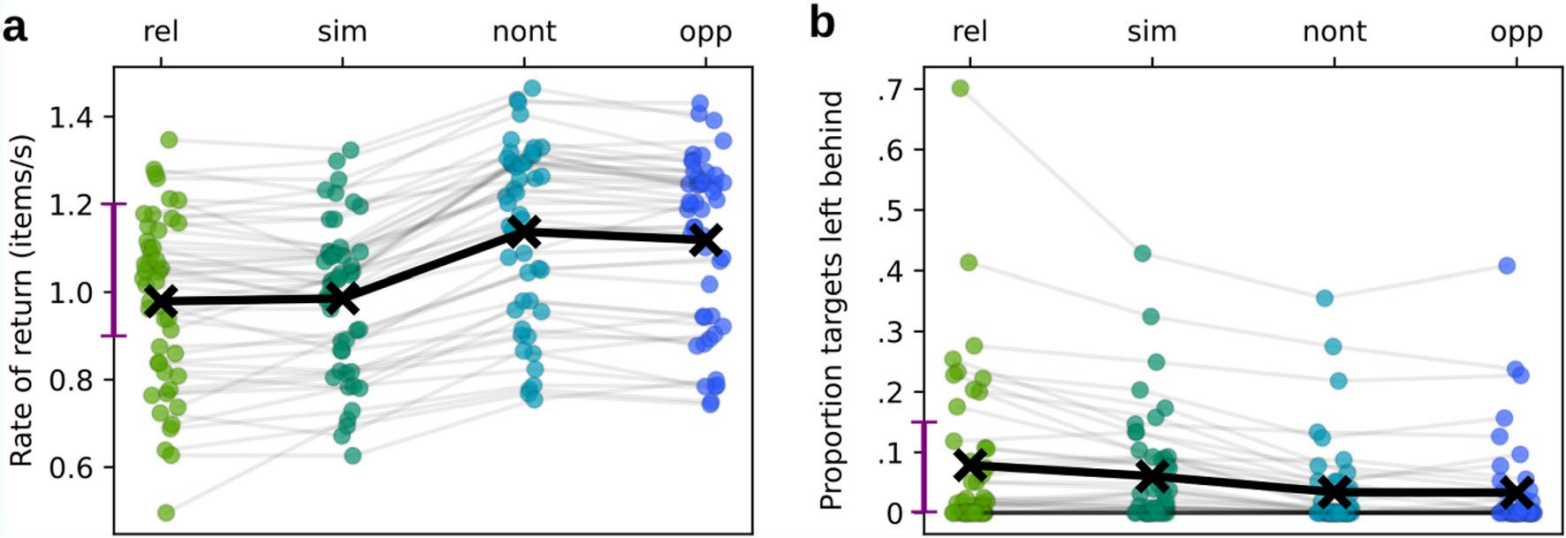
Descriptive plots of the data. (**a**) Average rate of return in the conditions, and (**b**) proportion of targets left behind in a patch; “rel”, “sim”, “nont”, “opp” refer to the distractor conditions Relational, Similar, Non-target, and Opposite. Each line of connected points represents the same participant in the different conditions. To give better impressions of the distributions (especially in (b), where many points are located at and near zero), the points were jittered and rendered semi-transparent. The × -markers and the bold black lines represent the means over participants. The purple bar on the y-axis indicates the ranges that are also represented in Fig. 4.

On average, participants took 5.4 blocks (*SD* = 1.04) to reach the goal of 1000 points. Each completed block contained 12 trials (3 of each condition); the last block was not necessarily complete because foragers could pass the point limit before completing it. Note that the blocking merely served to balance the conditions over the course of the experiment and is not further considered in the analyses. Foragers rarely made any mistakes. The error rates (percentage of selections that were no targets) was lower than 0.8% for all conditions, and a one-way Bayesian repeated measures ANOVA (on the arcsine-transformed proportion data) returned a BF_incl_ = 0.082 (according to which it is 12 times more likely that there is no meaningful difference in error rates). Consequently, error data was not further analysed. Figure 3 shows descriptive plots of the main variables of interest, rate of return and the proportion of targets left behind.

### Rate of return

**Figure 4:**
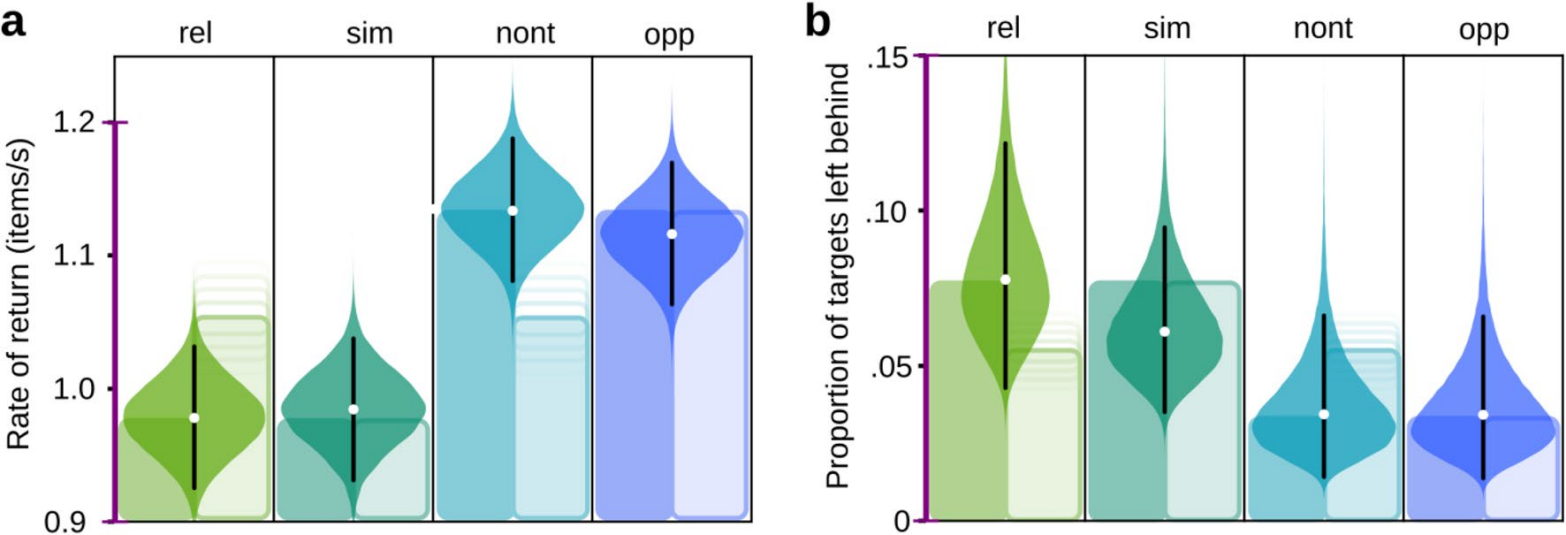
Model estimates of the central tendencies on the group level. (**a**) Average rates of return in the conditions, and (**b**) average proportions of targets left behind in a patch; “rel”, “sim”, “nont”, “opp” refer to the distractor conditions Relational, Similar, Non-target, and Opposite. The distributions are the posteriors estimated with Bayesian hierarchical models. The white dots indicate the means, and the black lines the 95% HDIs. The bar graphs in the background illustrate the order predicted by the two competing hypotheses (and are scaled to the observed minimum and maximum). Note, that as in Fig. 3, the intermediate distraction predicted by the feature-specific account can be anywhere between low and high. This is indicated by the smearing of the vertical boundary of the intermediate bars. Solid bars refer to the relational account, faint bars to the feature-specific account. The purple bar on the y-axis extends over the same range as the corresponding purple bars in Fig. 3.

The rate of return (RoR) in items per second is the number of items collected in a patch divided by the time spent in the patch (plus the “travel time”, in our case a fixed 1 s duration). The RoR hence reflects the performance in terms of speed with which participants forage in a condition. Figure 4a depicts the group-level posteriors of the central tendencies for the four different conditions estimated with a hierarchical Bayesian model (see Supplementary Methods). The Relational and Similar distractor contexts both led to low rates of return of roughly one item per second (RoR_rel_: 0.978 [0.922, 1.033]^HDI95^; RoR_sim_: 0.985 [0.929, 1.040]^HDI95^), the difference RoR_rel_ − RoR_sim_ is only −0.007 [−0.020, 0.007]^HDI95^, with zero, no difference, within the 95% HDI. Similarly, Non-target and Opposite distractor contexts both have comparable rates of return (of about 1.1 items per second; RoR_nont_: 1.136 [1.08, 1.192]^HDI95^; RoR_opp_: 1.117 [1.061, 1.171]^HDI95^). The difference RoR_nont_ − RoR_opp_ was estimated at 0.020 [0.005, 0.033]^HDI95^ with zero, no difference, just outside the 95% HDI. The Opposite and Non-target rates are higher than the Relational and Similar rates (RoR_opp_ − RoR_sim_, the smallest difference of the possible combinations is 0.131 [0.118, 0.145]^HDI95^), with zero clearly outside the 95% HDI. This pattern agrees with the prediction of the relational account (cf. Fig. 4a, solid bars). When the model is constrained to only allow the relative differences indicated in these patterns (e.g., for the relational account “rel” and “sim” must be equal and both low, and “opp” and “nont” must be equal and both high), the two accounts can be tested directly against each other in a formal model comparison. We calculated scores based on Pareto-smoothed importance sampling leave-one-out cross-validation^25^. The scores are listed in Table 1 and they reflect that the relational account’s prediction outperforms the feature-specific version. The weight listed in the table (which can be loosely interpreted as the probability of each model) is 1.00 for the relational version and a weight of 0.00 for the feature-specific version (see Methods section: Model Comparisons for a descriptions of all the scores).

**Table 1:** Results of the model comparison based on the rate of return.

Model	rank	loo	p_loo	d_loo	weight	se	dse
Relational	0	1894.11	142.84	0.00	1.00	47.18	0.00
Feature specific	1	1602.17	176.89	292.93	0.00	4719	22.2

Lower rank corresponds to preferable model. Column “loo”: Score of the Pareto-smoothed importance sampling leave-one-out cross-validation. Column “p_loo”: Estimated effective number of parameters (higher for more complex models). Column “d_loo”: Relative “loo” difference to the top ranked model. Column “weight”: Weight can be loosely interpreted as the probability of each model. Columns “se” and “dse”: standard errors of the loo scores and relative differences, respectively.

### Targets left behind

**Figure 5:**
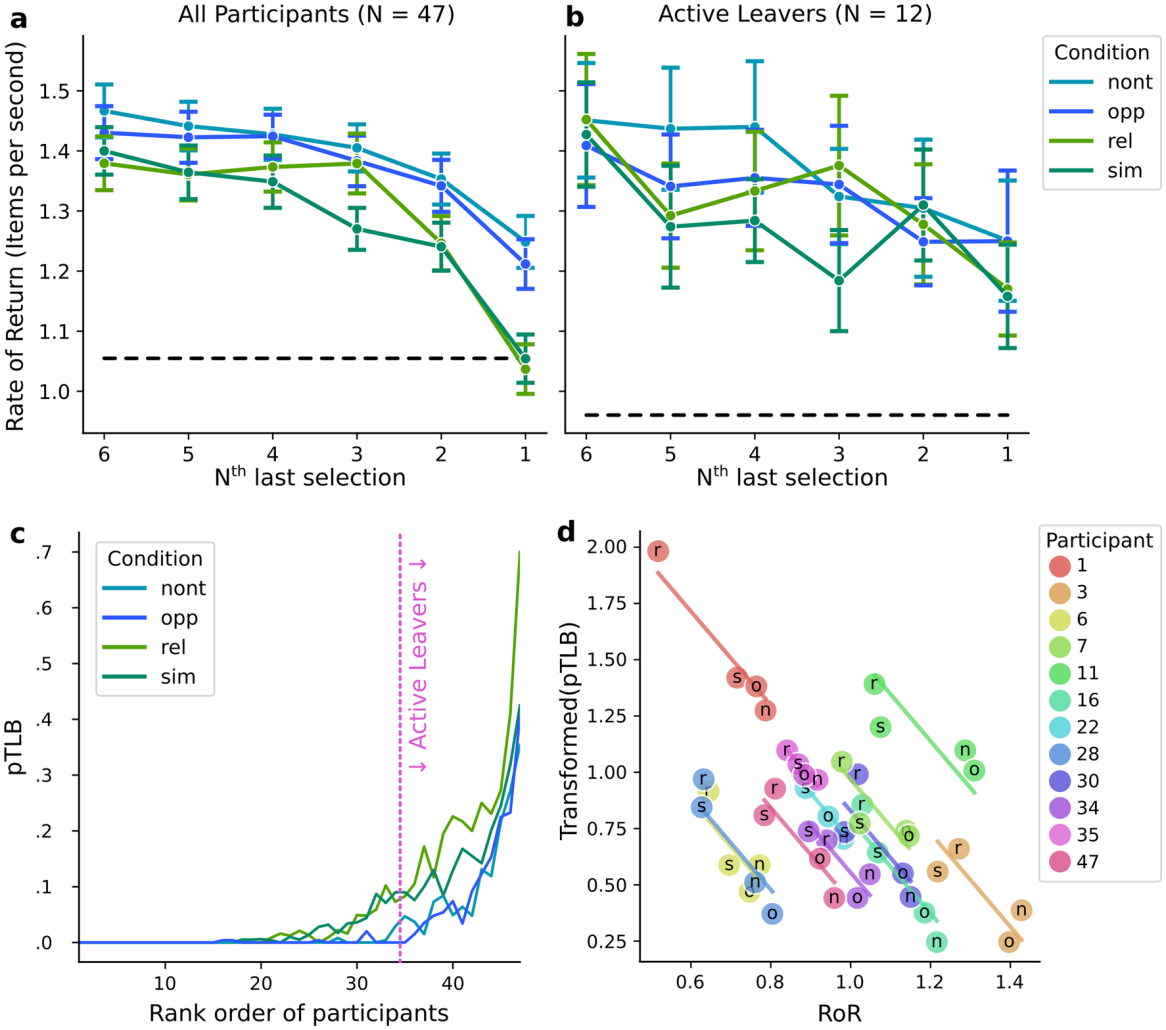
Patch leaving behaviour. (**a**) Development of the instantaneous rate of return over the last six target selection clicks in a trial for each condition (mean over participants, error bars indicate the standard error of the mean). The black dashed line indicates the average rate of return in the experiment (based on collecting in the patches and travel time between patches). (**b**) The six last target selections as in (a) but only for participants who actively left the patches. (**c**) The proportion of Targets left behind (pTLB) of individual participants ordered after increasing pTLB summed across all conditions (rank order of participants). The pink dashed line represents the split into participants that left at least one target behind in each condition, leaving the patch actively (right of the line), and those who did not (left of the line). (**d**) Repeated-measures correlation of pTLB (arcsine-transformed) and the Rate of return (RoR) only for active leavers. The letters inside the markers refer to the four conditions. In (a to c) “rel”, “sim”, “nont”, “opp” refer to the distractor conditions Relational, Similar, Non-target, and Opposite. In (**d**) only the first letter of the condition name is used to identify the condition. Best viewed in colour.

Observers might leave different amounts of targets behind in the different conditions when they leave a patch to move to a new one. Overall, foragers left rather few targets behind, many participants collected all targets in each patch in all conditions (cf. Figs. 4b and 5c), leading to distributions with peaks near zero and only a few larger values. To obtain estimates of the central tendencies for each condition, we employed a hierarchical beta-binomial model^26^ that can take these asymmetries into account to estimate group-level and participant-level proportions of targets left behind (pTLB). The group-level posterior distributions for the four conditions are plotted in Fig. 4b and we report pairwise comparisons below. In the Relational distractor condition the proportion of targets left behind is the highest (pTLB_rel_: 0.072 [0.043, 0.125]^HDI95^). It differs from all other conditions (pTLB_rel_ − pTLB_sim_: 0.018 [0.013, 0.024]^HDI95^; pTLB_rel_ − pTLB_nont_: 0.045 [0.04, 0.049]^HDI95^; pTLB_rel_ − pTLB_opp_: 0.045 [0.041, 0.05]^HDI95^). In the Similar distractor condition the estimate is somewhat smaller (pTLB_sim_: 0.058 [0.034, 0.096]^HDI95^) but foragers are more likely to leave targets behind than in the Non-target and Opposite conditions (pTLB_sim_ − pTLB_nont_: 0.026 [0.022, 0.031]^HDI95^; pTLB_sim_ − pTLB_opp_: 0.027 [0.023, 0.032]^HDI95^). The Non-target and Opposite distractor conditions show the lowest proportions of targets left behind (pTLB_nont_: 0.030, pTLB_opp_: 0.029). The difference between them is virtually zero (pTLB_opp_ − pTLB_nont_: − 0.001 [**− **0.004, 0.003]^HDI95^).

The bars in the background of Fig. 4b illustrate the patterns predicted by the relational and feature-specific accounts. For a formal model comparison between the two alternatives, leave-one-out cross-validation scores are listed in Table 2. The relational account model outperforms the feature-specific model. The weight is 0.74 for the relational and 0.26 for the feature-specific account. The reason for these weights being somewhat less distinctive than the ones for the RoR is likely that neither the relational nor the feature-specific account predicts that the Similar distractor context leads to an intermediate proportion of targets left behind (see Fig. 4b, “sim”, where the result matches neither prediction bar).

**Table 2:** Results of the model comparison based on the proportion of targets left behind. Lower rank corresponds to the preferable model.

Model	rank	loo	p_loo	d_loo	weight	se	dse
Relational	0	− 3142.04	109.87	0.00	0.74	93.9	0.00
Feature specific	1	− 3298.09	159.01	156.05	0.26	97.83	30.77

Column “loo”: Score of the Pareto-smoothed importance sampling leave-one-out cross-validation. Column “p_loo”: Estimated effective number of parameters (higher for more complex models). Column “d_loo”: Relative “loo” difference to the top ranked model. Column “weight”: Weight can be loosely interpreted as the probability of each model. Columns “se” and “dse”: standard errors of the score and relative difference, respectively.

### First vs. second selection

**Figure 6:**
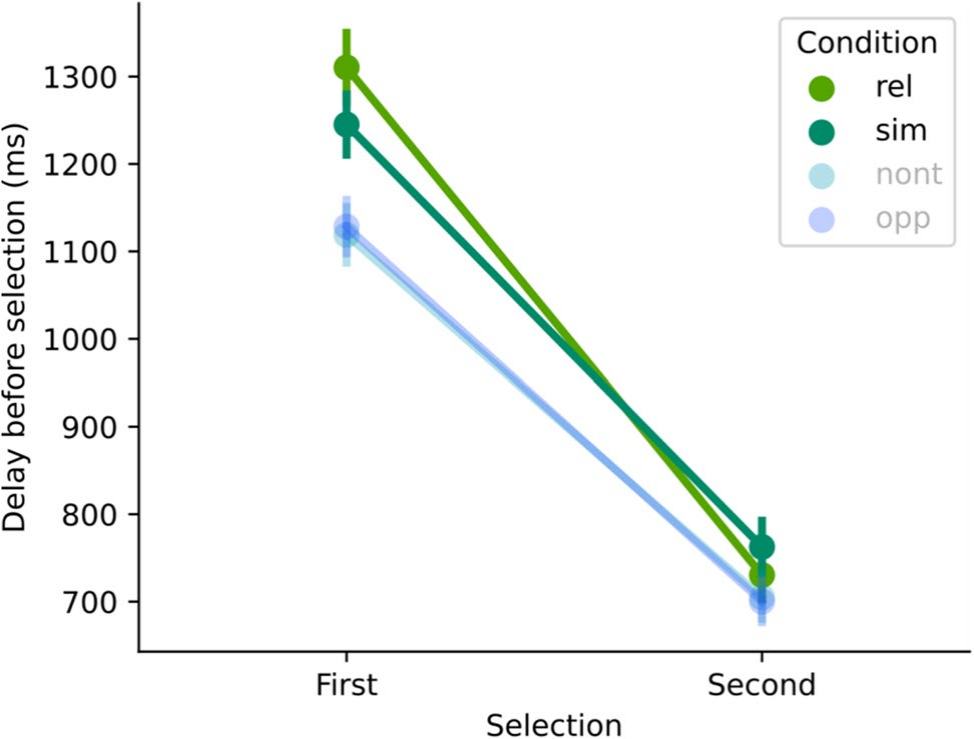
Comparisons of delays before the first selection (time since trial start) and second selection (time since the first selection) for the Relational (rel) and Similar (sim) condition. For completeness, the Non-target (nont) and Opposite (opp) conditions are also plotted (with fainter colours). Best viewed in colour.

To test whether the relational distractor might have a particularly strong impact at the start of the trial (cf. Hamblin-Frohman et al.^24^), we compared the delay before the first selection (time since trial start) and second selection (time since the first selection) between the Relational and Similar condition (see Fig. 6).

A two-by-two repeated measures ANOVA with the factors Selection (“First”, “Second”) and Condition (“rel”, “sim”) provided support for the interaction of selection and condition (BF_incl_ = 462.72; the main effect for selection received a BF_incl_ = 3.8 × 10^27^ and condition a BF_incl_ = 0.308). Bayesian directed (“rel” > “sim”) paired *t* tests confirmed the longer delay before the first selection (BF_+0_ = 17.1; and there was evidence against such a difference for the second selection: BF_0+_  = 17.7).

### Patch-leaving behaviour

To assess whether our participants left patches in agreement with the MVT or whether there are deviations that might depend on the relational properties within the patches, we analysed the patch-leaving behaviour (see Fig. 5). As expected, the instantaneous rates of return declined toward the last selection in a patch, as elements in the display got rarer. The instantaneous rate of return is the rate (in items per second) with which a forager collects points at any given moment, calculated as one divided by the inter-target time (time since the previous selection). A Bayesian repeated measures ANOVA confirmed this main effect of selection number (BF_incl_ = 1.9 × 10^41^). Moreover, a main effect of distractor condition was detected (BF_incl_ = 6.68 × 10^10^) and evidence for an interaction of distractor condition and selection number (BF_incl_ = 18.18). Post hoc comparisons of the distractor conditions showed that Relational and Similar differed from Opposite and Non-target (BFs of all combinations were higher than BF_10_ = 7.58 × 10^4^). The conditions Relational and Similar had similar rates of return (BF_01_ = 7.04 in favour for the rates being the same) as had Opposite and Non-target (BF_01_ = 2.67). To test whether observers foraged “optimally” in agreement with the MVT, we performed paired Bayesian *t* tests of the instantaneous rate of return at the last selection in a patch against the average rate of return in the experiment. Note that even though we have four different distractor conditions, we test the instantaneous rates of them against the same average rate of return across all conditions because trials from all conditions were presented randomly intermixed, and foragers could not expect to enter any particular condition when leaving a patch for the next. The test revealed that in the Relational and Similar conditions foragers indeed left the patch when they met the average rate of return (BF_01_ = 5.43 and BF_01_ = 6.33 in favour of a match between the average rate of return and the instantaneous rate at the last click). In the Non-target and Opposite condition, the instantaneous rates of return at the last selections were far higher than the average rate (cf. Fig. 5a; BF_10_ = 1.14 × 10^7^ and BF_10_ = 8.84 × 10^3^, in favour of a difference).

A large portion of the participants always collected all targets (see Fig. 5c). Consequently, their last selection click in a trial might not result from a decision based on their rate of return but might merely reflect the fact that all targets were depleted. To provide a better view on the decisional factor, we isolated a subsample of participants that left at least some targets behind in every condition and actively left the patches (Fig. 5c, “Active Leavers”). For these 12 active leavers, we performed the same analyses as described above and plotted the development of the rate of return in Fig. 5b. The Bayesian repeated measures ANOVA again showed the main effects of selection number (BF_incl_ = 9.09 × 10^5^) and distractor condition (but only with a small BF_incl_ = 2.25) and neither substantial evidence for nor against an interaction of these two factors (BF_incl_ = 0.91). In this smaller subsample, the pattern of differences between the distractor conditions is less clear. Only the difference between the Similar and Non-target conditions was supported by a substantial Bayes factor (BF_10_ = 207.32) and the Bayes factors of all other pairwise comparisons were rather inconclusive (BFs_10_ between 0.14 and 2.25). As can be seen in Fig. 5b, the last selection clicks in this subsample were clearly above the average rate of return, which was supported by Bayesian *t* tests (Relational: BF_10_ = 187.2 Similar: BF_10_ = 17.9, Non-target: BF_10_ = 88.84, Opposite: BF_10_ = 8.09).

For the active leavers, we also correlated their rate of return with the proportion of targets left behind (arcsine transformed), as shown in Fig. 5d, to provide a general check of whether participants conducted some form of optimal foraging. The repeated measures correlation revealed a negative correlation of *r(35)* = ** − **0.88, *p* < 0.001. Note, that the scatter plot in Fig. 5d suggests that this correlation is mainly driven by the two less efficient conditions (Relational and Similar) clustering at higher proportions of targets left behind and lower rates of return and the two more efficient conditions (Non-target and Opposite) clustering at lower proportions of targets left behind and higher rates of return.

## Discussion

The present study shows that foraging behaviour is impaired by relational and target-similar distractors to similar degrees. Both types of distractors match the relational properties of the target (e.g., like the targets, both are “greener” than the non-targets). The effect is predominantly revealed in the rate of return, which quantifies the efficiency with which participants forage. Our model estimates show that the rate of return is lower in the presence of Relational and (Target)-Similar distractors (to similar degrees) and higher in the Non-target and Opposite conditions. This pattern is well in agreement with the relational account of attention guidance^12^ and deviates from predictions of a purely feature-specific account, which assumes that the target-similar distractor leads to higher distraction and less efficient foraging than the distractor that differs from the target features but matches its relational properties. These conclusions are also supported by a formal model comparison: the relational model outperforms the feature-specific model when a model that implements the predicted pattern of the relational account is tested against one that implements the feature-specific predictions.

We also looked at the number of targets foragers left behind in the different conditions. If foraging was influenced by the relational properties, they should have left more targets behind in the Relational and the Similar conditions compared to the Non-target and Opposite conditions. The results agree with this prediction, with only one exception: slightly fewer targets were left behind in the Similar compared to the Relational condition. This small but unexpected difference might hint that attention guidance comes with additional cognitive effort in the presence of relational distractors. In the presence of such distractors cognitive effort seems to arise which does not lead to lower RoRs but which foragers might try to avoid by leaving patches earlier. When considering the feature-specific account, the data pattern of targets left behind matches the prediction that the Opposite distractor condition should lead to the lowest level of distraction but deviates from the predicted pattern in the other three conditions. Therefore, even though not perfectly, the relational account seems to explain the pattern of targets left behind better than the feature-specific account. This was confirmed by a formal model comparison in which the relational model outperformed the feature-specific one. In sum, attentional guidance in visual foraging seems to depend on relational templates.

Some result patterns of other recent foraging studies seem to agree well with relational guidance in foraging, as for instance foraging data from Tünnermann et al.^27^, who investigated how the feature context in search arrays influences switching between templates when two different target types are present. The highest reluctance to switch between two target templates and the least efficient foraging was found when a distractor was placed close to and in between the two targets in feature space. This could have discouraged relational strategies, effectively enforcing more effortful feature-specific search. Moreover, possible relational guidance was observed in a study by Li et al.^28^. Their patches contained T-shaped distractors, and ⅂-shaped targets (presented in random cardinal orientations), whose smaller strokes varied in offset between a maximum (⅂) and a minimum that was almost a perfect T. Participants of the study did not pick the different offsets evenly, nor did they prioritize targets with average offsets (that could potentially represent the class of target items best). Instead, they initially preferred the items with the largest offsets (maximally shifted away from the distractor context) and, over time, turned to items with decreasing offsets as would be predicted by relational guidance. This experiment was not designed to look at relational guidance, and other explanations (such as similarity with a prototypical L shape) cannot be ruled out, but the results are at least compatible with the idea of relational guidance.

Interestingly, in the present study, the pattern of search performance reflected in the rate of return (similar to the pattern reported by Martin & Becker^14^) is characteristic for a strict relational account because only the direction of the distractor feature difference mattered with respect to the (non-target) context but not the magnitude of the difference: Performance was the same in the Relational and Similar conditions, which both had the same relational difference (e.g. both “greener” than the environment) but differed in magnitudes (with the Relational distractor being, e.g., even “greener” than the Similar one). Various other studies reported patterns that only agree with a softened relational account that allows templates to have the relational character but also take the magnitude of the feature-distance into account. Some studies find that all distractors that match the relational properties attract attention rather similarly but with a tendency of declined attraction for distractors with features farther away from the veridical target features (cf. Yu et al.^18^; Hamblin-Frohman & Becker^13^). That is, “greener” can become “too green” or “not green enough” at some point. A relational template that declines with distance from the veridical target features could arise from a combination of relational and feature-specific guidance. For instance, the optimal tuning theory posits that a feature-based template is shifted away from the non-targets (but unlike a relational template has a defined extent in the feature space) to improve the signal-to-noise ratio by reducing feature overlap (see Navalpakkam & Itti^2^ and Kerzel^29^). Moreover, as another source that explains effects that look like mixtures or relational and feature-specific templates, both relational and feature-specific templates could be at work in different stages of processing (cf. Ort & Olivers^30^). Yu et al.^18^ suggest that the guiding template, which is employed in early attention guidance is purely relational, whereas the target template, which is used in the decision stage to confirm if previously selected candidates match the target uses feature-specific mechanisms (potentially somewhat shifted by optimal tuning).

There is also ample evidence in the literature for situations in which distractors that matched the relational properties best (“the greenest”) had the strongest impact. In other words, distractors that differed from the context in the same feature direction as the target and with the greatest magnitude hindered performance the most. This pattern seems to be present in studies that looked at eye movements early in the trial: Martin and Becker^12^ , Becker et al.^14^, Yu et al.^16^, and Hamblin-Frohman et al.^23^ found higher proportions of first fixations to relational distractors with the largest feature-space distance compared to distractors with the same relational properties but smaller feature-space distance. As mentioned above, we did not find such “super-relational” effects where the direction matters but also the magnitude with which the distractor is shifted in that direction in the foraging efficiency when we looked at average performance over the whole trial (see Fig. 4a.) or at the end of a trial when foragers had experienced the feature context for the longest time (Fig. 5a and b, last selections). However, since these effects were reported mainly for early saccades, they could occur very early in the trial, perhaps when the visual system still has to adjust control settings to the new feature context. In this case, the effect is most likely only measurable in foraging of the first element in a patch and would probably be masked in the average over many selections, most of which might not be affected by it. To this end, we analysed the delay before the first and second selections in the patch and compared the Relational and Similar conditions. We indeed found a super-relational effect at the beginning of a trial as a larger delay in the Relational condition for the first selection which then disappeared for the second selection (see Fig. 6). Hence, this is in line with the earlier studies mentioned above but suggests that the effect is very short-lived and does not determine behaviour when observers interact with the environment for longer durations. Importantly, this does not imply that relational guidance disappears over time. To the contrary, as outlined above distraction from “strict relational guidance” (in the sense that only the direction and not the magnitude of the difference matters) remains present until observers leave the patches.

In the present study we were also interested in how the relational properties of the distractor conditions affect strategic decisions in foraging. Earlier research has shown that humans compare their momentary efficiency (their instantaneous rate of return) against the average rate of return in the environment, and leave the current patch if the performance drops below this average (e.g., Wolfe^23^; Cain et al.^31^; Ehinger & Wolfe^32^). Our analysis of patch-leaving behaviour showed that observers not always stayed within the patches until their performance dropped to the average rate of return. In the Non-Target and Opposite conditions (where foraging was most efficient) patches were left when instantaneous rates of return were still high above the average. In the Relational and Similar (the less efficient) conditions, patches were left when the rates just reached the average. However, this pattern might be distorted by the fact that the majority of the participants collected all the targets in the patches and hence did not leave the patches by choice but because no more targets were present (and the patch ended automatically). This raises two questions: (1) Did those foragers who *not* always collected all targets behave in agreement with the marginal value theorem, and (2) did those who collected all targets perform in agreement with optimal foraging theories in general? To shed light on these questions we also analysed the patch-leaving behaviour of the subset of participants that left some targets behind in each of the conditions. Results showed that these “active leavers" left patches too early with their instantaneous rates of return still high above the average rate of return. This suggests that in our experiment active patch-leaving led to “less optimal” performance compared to foragers who collected all targets in a patch, and that active leavers did not behave in agreement with the marginal value theorem. However, staying until all targets were collected was the best foragers could do, in general agreement with optimal foraging theory. That foragers generally aimed to increase their time efficiency by leaving disadvantageous patches early can also be seen in the substantial correlation between the proportion of targets left behind and overall rate of return per trial: In conditions where foraging was less efficient (Relational and Similar) the active leavers left earlier than in the conditions with more efficient foraging (Non-target and Opposite; cf. Fig. 5d). That foragers who used active patch leaving to improve their rate of return still left patches too early could be due to different reasons: They may not have been able to accurately monitor the momentary rate of return, estimate the average rate of return (perhaps due to their self-timed breaks), or other factors might have triggered them to leave patches. One factor that differed from many other visual foraging experiments was that the stimuli were in constant motion. Under such dynamic conditions, observers can employ sit-and-wait strategies (cf. von Mühlenen et al.^33^) to some degree, where they focus their search on particular areas and “wait” for new targets to arrive. Foragers who prefer such strategies might move to new, more busy patches (in which sit-and-wait is a more promising strategy) too early to avoid switching to a more active strategy, even though it leads to suboptimal performance in terms of time spent foraging (it might, however, reduce energy expenditure).

In conclusion, the present study showed that relational attention guidance not only arises in highly artificial tasks but also in visual foraging tasks which capture many aspects of natural visual search, such as search for and action planning toward multiple targets over longer behaviourally continuous periods. This strengthens the account that search templates encode relational properties and hints that relational guidance is relevant beyond the lab.

## Methods

### Participants

An international sample of 50 participants was recruited on the online platform Prolific (www.prolific.co). This sample size was based on the earlier experiences with the foraging paradigm and similar models (see Supplementary Method: Sample Size for details). The experiment lasted approximately 30 min, for which participants were paid ₤3.6 Sterling. As an incentive to collect items quickly and without errors, the fastest five participants were paid a bonus of ₤2. Two participants were excluded from the analyses due to extremely high numbers of clicks (> 600) on non-targets and distractors, which led to the conclusion that they did not seriously engage in the task, did not understand the instructions correctly, or had technical issues. The dataset of one person included 23 inter-target times (ITT; the delay between two collection clicks) smaller than 30 ms (the average ITT of the other participants is 881 ms), which could only occur due to technical issues or fraudulent technical clicking aids. This participant was excluded as well (see Supplementary Methods: Removal of three participants). The remaining 47 participants were included in the analyses. All participants (16 females, 31 males; mean age = 25 years, range 18–57 years) confirmed that they had normal or corrected-to-normal visual acuity and good English proficiency. Participants gave their informed consent. All procedures were approved by the ethics commission of the Faculty of Psychology at Philipps-University Marburg as part of an experimental series on visual search.

### Apparatus and stimuli

The stimuli were small disk and square shapes which were presented on a light grey background (CIELAB: 89.53 0.00 0.00). The display was specified in a resolution of 1920 × 1080 pixels, which was stretched to fill the entire screen (but keeping the aspect ratio intact). The actual extent in degree of visual field varied depending on the screen sizes and viewing distances of the participants. On a typical 24″ (53.13 cm × 29.89 cm) widescreen monitor at a viewing distance of 60 cm the area extended over 47.76° by 27.97°. The measures in degree given below refer to this exemplary setup. Each patch consisted of 90 stimuli: 15 targets, 15 distractors, and 60 non-targets. Start positions of stimuli were randomly distributed on a centred grid with 11 rows and 20 columns. The grid cells had a spacing of 90 pixels (2.38°) vertically and horizontally, and a random jitter in the range of ± 40 (1.06°) pixels was applied, leading to a total width and height of the arrangement of approximately 1830 × 1020 pixels (45.76° × 26.47°). Stimuli immediately started to move randomly across the screen with a speed of 30 pixels per second (0.79°/s). Their initial direction was selected at random. Consequently, stimuli could briefly overlap when they crossed one another. When they crossed the borders of the screen, stimuli “turned” and moved in a new random direction that brought them back into the display. The word “Next” (in grey; CIELAB: 42.37 0.00 0.00) was shown in the upper right corner and could be clicked to leave a patch and move to the next.

The colours of the stimuli were the same as the ones used by Martin and Becker^14^. The four colours, “green”, “olive”, “aqua”, and “blue”, reside in the green-to-blue range of the colour wheel. The neighbouring colours were equidistant in the CIELAB colour space. Targets were either aqua- (CIELAB: 49.55 −23.44 −23.50) or olive-coloured (CIELAB: 50.68 −40.51 11.06) squares (height and width 27 pixels; 0.71°) or disks (diameter 30 pixels; 0.79°) with both shapes covering an area of approximately 707 pixels^2^ (18.52°^2^). Non-targets always had the same shape as the targets but were olive when the target was aqua and aqua when the target was olive. The non-targets accounted for by far the largest share of all stimuli in the display, and therefore determined the relevant context for the search. Distractors always had the other shape than targets and non-targets: when targets and non-targets were squares, distractors were disks and vice versa. The distractor colour varied depending on the condition. In the “Relational” condition the distractors were green (CIELAB: 41.32 −43.77 44.71) for olive targets and blue (CIELAB: 50.19 15.67 −77.96) for aqua targets. In the “Similar” condition, distractors had the same colour as the targets. In the “Non-target” condition, distractors had the same colour as the non-targets. In the “Opposite” condition, distractors were green for aqua targets and blue for olive targets.

As participants did the experiment online, the luminance and the exact colour presentation of the stimuli are unknown. To ensure that colours were shown in the best way possible, participants were instructed to turn screen brightness to maximum and deactivate night-mode. To counter possible imbalances in how consumer-grade displays render these colour ranges, the roles of the target and non-target colours (and consequently the distractor colours) were varied between participants.

### Design and procedure

The experiment consisted of four conditions in a within-subject design, where the colour of the distractors varied as described above. Within one block, all four conditions were randomly repeated three times, resulting in 12 trials in each block. Every participant was randomly assigned to one target colour-condition, so that about half of the participants collected aqua-coloured targets and about half collected olive-coloured targets for the whole experiment (including practice trials). Every two blocks the target shape switched.

The participants’ task was to collect 1000 points as fast as possible by clicking on targets with the mouse. For each collected target, participants gained one point. A collected target disappeared. Non-targets and distractors were not collectable and shrunk to 20% of their original size when clicked on (growing back to normal size within 50 ms) resulting in a quick flicker. Clicking on non-targets or distractors did not subtract or add any points. Participants could leave each patch whenever they wanted with a travel time (to the next patch) of 1000 ms by clicking on the “Next”-button in the upper right corner of the display. When all targets were collected, the next patch was presented automatically after 1000 ms. Before the start of the experiment four practice trials were included. At the beginning of each block, the object that had to be collected was shown. Participants started the blocks by clicking on a button. Between the blocks, they could take small breaks and continue when ready. After each block, a feedback screen showed the current point score and the collection speed (targets collected per minute) in the previous block. In later blocks, also the collection speed of the second to last block and the third last block were shown. After each patch the experiment checked whether participants had reached or surpassed 1000 points and the experiment ended if this was the case.

### Data analysis

#### Outlier removal

Given that the experiment was conducted remotely in the participants’ web browsers, several measures were applied to ensure good data quality: The 47 datasets underwent a filtering that detected trials with longer interruptions (more than 10 s at the beginning of a trial or 20 s between selections). Then a model-based algorithm was applied to remove strong outliers in the patch leaving strategy. These might occur due to spontaneous short-lived strategy changes or accidental clicks on the “next patch” button which are not representative for the overall strategy of a person in a given condition. Further details on the rationale behind these steps and on their implementation can be found online in the Supplementary Method. Importantly, note that despite the fact that there are multiple criteria and multiple steps, less than 2.04% of the trials were removed in this procedure.

#### Hierarchical Bayesian models (HBM)

The main variables of interest (Rate of return and targets left behind) were assessed with Hierarchical Bayesian parameter estimation (see Kruschke and Lidell^34^), which allows to estimate foraging parameters on the participant- and group-level in a coherent approach. Moreover, model comparisons can be performed. The models were implemented in PyMC^35^ and the model structure is described below for each variable. 20,000 samples (and 2000 tuning samples) were drawn using the NUTS sampler (see Hoffmann and Gelman^36^). For textual report, the modes of the posteriors are reported as point estimates and their 95% Highest-Density Interval (HDI^95^) as measures of credibility_._ If the HDI^95^ of a difference of interest (e.g., between the estimates for two different experimental conditions) does not include zero, a null effect (no difference) is highly unlikely (cf. Kruschke and Lidell^34^). Note, however, that 95% is an arbitrary choice and no hard cut-off: gradually less certain differences simply provide gradually less evidence for effects. The priors were chosen to be very vague so as not to distort the outcomes. Importantly, for the free-parameter model (which was used for parameter estimation), the same prior distributions were applied to all four conditions. The effective priors on the group-level central tendency parameters are visualized and discussed in Supplementary Method: Visualisation of the prior.

#### HBM: Rate of return (RoR)

The overall rate of return achieved in trials of the different conditions (RoR) was quantified via a hierarchical Bayesian model with a Normal likelihood, as the RoR varies continuously. For parameter estimation, the mean RoR were allowed to vary freely. For model comparisons (Pareto-smoothed importance sampling leave-one-out cross-validation, see Vehtari et al.^25^), the RoR means were constrained to vary relatively to each other in agreement with the predictions from either the relational or feature-specific account. Further details on model structure and the prior implementation can be found online in Supplementary Method: Model structures and priors, RoR. The effective prior on the central tendencies on the group-level is visualised and discussed in Supplementary Method: Visualisation of the prior.

#### HBM: Proportion of targets left behind (pTLB)

The proportion of targets left behind (pTLB) when leaving a patch is modelled with a Bayesian hierarchical beta-binomial model (appropriate for count data, cf. Albert and Hu^26^, pp. 381–385; Tünnermann et al.^27^), following the same logic with regard to parameter estimation and model comparison as described above. Further details on model structure and the priors can be found online in Supplementary Method: Model structures and priors, TLB. The effective prior on the central tendencies on the group-level is visualised and discussed in Supplementary Method: Visualisation of the prior.

#### Model comparisons

For comparisons between a relational and a feature-specific model, we implemented versions of the HBMs described above, which restricted the order of the parameter magnitudes as illustrated in Fig. 4a and b (see Supplementary Method: Model structures for details on the implementation). These model versions were compared using leave-one-out cross-validation approximated with Pareto-smoothed importance sampling based on the model posteriors^25^ as implemented in ArviZ^37^. That is, the procedure estimates how well the model would predict data points if the model is fitted without them. The procedure returns several informative scores: "rank" is a ranking of the models included in the comparison, with lower ranks referring to better models; "loo" is a score that determines this ranking (it is the log pointwise predictive density), where larger values refer to better model fit; the "p_loo", the estimated effective number of parameters, that quantifies the model complexity (which is taken into account when estimating the loo score; effectively, models are penalized for unwarranted complexity); “d_loo” is the estimated difference between the loo scores; the “weight” roughly corresponds to the probability of the models (more formally, it is the weight one should apply to each model if they are used in model averaging, that is, when both models are used in a weighted combination to predict new data); “se” is the estimated standard error of the loo score and “dse” is the standard error of d_loo.

#### Further analyses

The analyses of the delays before the first and second selections, and the analyses of the patch-leaving behaviour were executed with JASP 0.17^38^. Bayesian repeated measures ANOVAs to which the data were provided as mean scores for each person in each condition were used. Priors were set to default with all included models being equally likely. Analyses of effects across matched models are reported, with the inclusion Bayes factors indicating the evidence for including the respective predictor in the model^39^. Post-hoc Bayesian *t* tests were performed.

The correlation of TRR and TLB was assessed using a repeated measures correlation approach^40^ implemented in the Pingouin Python package^41^. Posterior modes of the participant-level estimates of TRR and TLB obtained with the models described above were used as point estimates to enter the correlation.

## Supplementary Information

Supplementary Information.

## Data Availability

The data and analyses scripts can be found at https://osf.io/54az9/.
