# Supplementary material for: Testing a relational account of search templates in visual foraging
Source: Sci Rep. 2023 Aug 2;13:12541. doi: 10.1038/s41598-023-38362-9 (PMC10397186; doi:10.1038/s41598-023-38362-9)
Supplement: Supplementary file 1 — Supplementary Information. [file 41598_2023_38362_MOESM1_ESM.pdf]

Supplementary Information

Testing a Relational Account of Search Templates in Visual Foraging

Inga Grössle, Anna Schubö, and Jan Tünnermann*

Cognitive Neuroscience of Perception and Action,
Department of Psychology, Philipps-University Marburg, Germany
* jan.tuennermann@uni-marburg.de

Supplementary Method

Sample size

In Tünnermann et al.[1] we computed that changes as small as approximately 0.1 to the switching probability between target types can be resolved with a Bayesian model with the 16 participants in that sample. In Tünnermann et al.[1], we conservatively chose to record twice as many participants (Experiment 1: 35, Experiment 2: 36). Both experiments reliably resolved small changes to the target switching probability (Experiment 1: at least as small as 0.05, see Figure 3, Experiment 2: at least as small as 0.1, see Figure 5). Experiment 2 showed that also subtle differences in the target choice probability (see Figure 7, last row) of about 0.03 could be revealed with this sample (Experiment 1 did not look at this). The binomial model of targets left behind in the present study is very similar to the switching/choice models used in Tünnermann et al.[1]. Hence, in the present study, we expected to resolve similarly small changes (0.1 roughly corresponds to differences of 1.5 targets left behind on average). Both experiments from Tünnermann et al.[1] also analysed the inter-target times (which are inversely proportional to the rate-of-return measure used in the present study). They found evidence for distractor context effects (weak evidence in Experiment 1, BF_{incl} = 2.78 but strong evidence in Experiment 2, BF_{incl} = 1.512E+22, where relational guidance might be the driving factor). Taken together, this suggests that sample sizes of 35 participants would provide enough power to observe the effects of different (relational) distractor contexts with sufficient precision. Since online data collection is quick but prone to drop-outs, we aimed to collect 50 participants to be on the safe side.

Removal of three participants

Because the experiment was conducted as a browser-based online experiment without direct control over the participants’ behaviour or their technical setup, we initially screened the data for obvious irregularities. Three participants produced data which indicated that they did not understand the task, did not engage in it seriously, or encountered technical problems with the

procedure: They clicked on 30 or more non-targets or distractors over the course of 12 respectively 6 trials. Making this many mistakes seem unlikely if participants performed the task as instructed and without technical issues. Their entire datasets were excluded from the analyses. One further participant was removed because their ITTs were implausibly low: 23 ITTs were smaller than 30 ms. This corresponds to clicking faster than 33 times per second whereas people typically click one to three times per second and a rate of 33 is likely impossible. The minimum ITT of this person was 6 ms, corresponding to more than 166 clicks per second. These values suggest that there was a technical problem or some fraudulent clicking aid was employed. Visualisations of the outliers can be found at https://osf.io/2nbtf.

Removal of trials with interruptions

Participants had been instructed to take breaks only between the blocks. However, given that the participants worked remotely on their own computers, interruptions during the trials (e.g., incoming e-mails, phone calls, etc.) are not unlikely. To detect such interruptions, we filtered the data concerning two criteria: (1) The time until the first selection in a display must not exceed 10 seconds and (2) none of the intervals between subsequent target selections must be larger than 20 seconds. These criteria are quite conservative (the mean time to the first selection was 1.2 seconds and the mean inter-target time was 0.89 seconds). The reason for the more lenient criterion on the inter-target times is that unlike as for the first selection, where the display is filled with 15 targets, inter-target times can get multiple seconds long if participants try to find the last (few) targets in a display. Even though this strategy is inefficient (leaving the patch would be the more efficient choice) the strategy is permissible and hence such trials should not be removed. Given that the described criteria only removed six out of 3,384 trials (0.18 %) and that these came from all conditions, we believe it removes the irregularities without distorting the results. The detailed procedure can be found at https://osf.io/92xkf.

Model-based removal of outliers

When calculating the leave-one-out cross-validation score via Pareto-smoothed importance sampling (PSIS)[2] on the dataset (after removal of the three participants and trials with interruptions, see above), diagnostics revealed that 35 observations (1% of the trials) had Pareto k values[2] larger than 0.7, flagged as “bad” or “very bad” (3,325 [98.4 %] flagged as “good”, 18 [0.5 %] as “OK”, 27 [0.8 %] as “bad”, and 8 [0.2 %] as “very bad”). This means these observations deviate so much from the other data and are so influential that they are not well predicted based on the remaining data in the cross-validation and the model comparison scores might not be reliable. We then removed all observations with $k > 0.6$ (i.e., with a slight margin to 0.7) and rerun the PSIS. We repeated this procedure iteratively until (after 6 iterations) only observations remained that were at least “OK” (3,302 [99.6 %] flagged as “good”, 13 [0.4 %] as “OK”, none as “bad” or “very bad”). The implementation of the algorithm and the log of the procedure can be found at https://osf.io/92xkf. In this procedure, a total 1.87 % of the trials that entered it was removed. As can be seen in Fig. S1, removals occurred in all conditions. The dataset resulting from this procedure was used in all analyses.

Note that it might not be a good idea in general to remove influential observations. However, as can be seen in Fig. S1, in the present case the removed observations often seem not to be meaningful with respect to the general strategy the foragers employed. Many of them might be caused by lapses and misclicks on the "next" button (perhaps when chasing a target moving close to it). For instance, participants 4, 5, 15, 26, 33, and 42 all only clicked the next button once in the whole experiment (in a trial the procedure ultimately removed) before they had collected all 15 targets like they did in the remaining 60+ trials. Since the removed trials are most likely no meaningful representations of the patch-leaving strategies, and since they only occur rarely and across all conditions, the removals are unlikely to bias our parameter estimates. However, in future work it might be good to prevent misclicks on the "next" button, and perhaps to amend the model with a parameter for strategy lapses or make it more robust with less conservative priors.

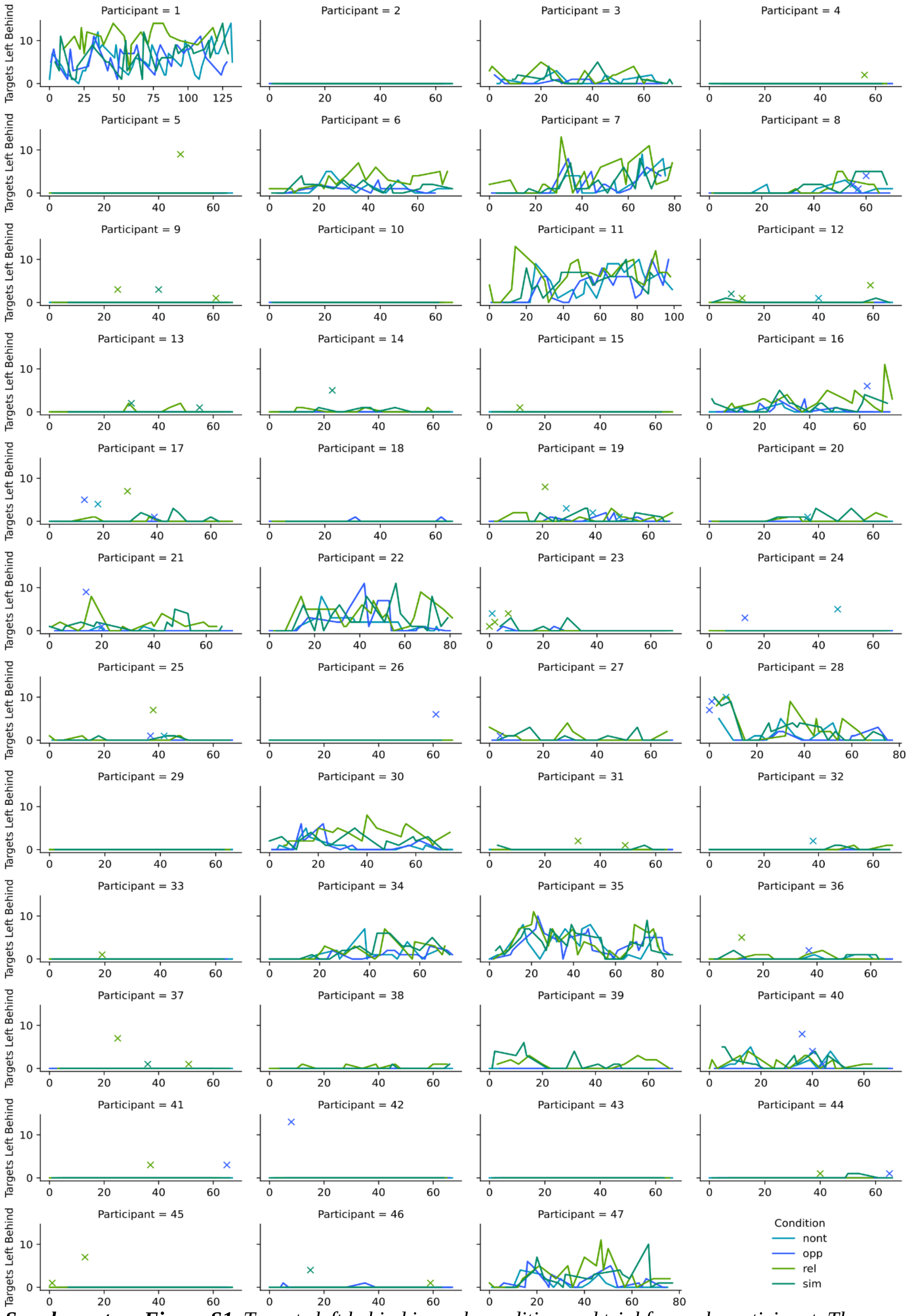

Supplementary Figure S1. *Targets left behind in each condition and trial for each participant. The "×" markers represent trials that have been deleted in the model-based removal of outliers.*

Model structures and priors

Rate of Return (RoR): The model structure and priors for the RoR models are listed below. All priors were chosen to cover large ranges of plausible values and, importantly, not give advantage to any condition. Variable $y_{i,j}$ refers to the RoRs observed for each participant *i* in each condition *j*:

Common part

$$\begin{aligned}
\mu_j^{\text{sd}} &\sim \text{HalfCauchy}(\sigma = 0.1)\\
\text{sd}_i &\sim \text{HalfCauchy}(\sigma = 0.5)\\
\mu_{i,j} &\sim \text{Normal}(\mu = \mu_j^{\text{mean}}, \sigma = \mu_j^{\text{sd}})\\
y_{i,j} &\sim \text{Normal}(\mu_{i,j}, \text{sd}_i)
\end{aligned}$$

Parameter estimation:

$$\mu_{j\in\{\text{rel,sim,nont,opp}\}}^{\text{mean}} \sim \text{Normal}(\mu = 1, \sigma = 0.5)$$

Model comparison (Relational):

$$\begin{aligned}
\mu_{j\in\{\text{rel,sim}\}}^{\text{mean}} &\sim \text{Normal}(\mu = 1, \sigma = 0.5)\\
\Delta\mu_{\text{high}} &\sim \text{HalfNormal}(\sigma = 0.15)\\
\Delta\mu_{\text{low}} &\sim \text{HalfNormal}(\sigma = 0.15)\\
\mu_{\text{high}(j\in\{\text{rel,sim}\})}^{\text{mean}} &\leftarrow \mu^{\text{mean}} - \Delta\mu_{\text{high}}\\
\mu_{\text{low}(j\in\{\text{nont,opp}\})}^{\text{mean}} &\leftarrow \mu^{\text{mean}} + \Delta\mu_{\text{low}}
\end{aligned}$$

Model comparison (Feature-specific):

$$\begin{aligned}
\mu_{\text{med}(j\in\{\text{rel,nont}\})}^{\text{mean}} &\sim \text{Normal}(\mu = 1, \sigma = 0.5)\\
\Delta\mu_{\text{high}} &\sim \text{HalfNormal}(\sigma = 0.15)\\
\Delta\mu_{\text{low}} &\sim \text{HalfNormal}(\sigma = 0.15)\\
\mu_{\text{high}(j\in\{\text{sim}\})}^{\text{mean}} &\leftarrow \mu_{\text{med}}^{\text{mean}} - \Delta\mu_{\text{high}}\\
\mu_{\text{low}(j\in\{\text{opp}\})}^{\text{mean}} &\leftarrow \mu_{\text{med}}^{\text{mean}} + \Delta\mu_{\text{low}}
\end{aligned}$$

The common part is used in both parameter estimation and model comparisons. For the parameter estimation, the mean RoRs of all conditions can vary freely and receive the same prior distribution (listed under "Parameter estimation"). For the models used in the model comparison (listed under "Model comparison (Relational/Feature-specific)"), the mean RoRs of conditions with expected low and high RoRs are defined relative (via the $\Delta\mu$ parameters) to the medium condition (or, for Relational, to a virtual midpoint between low and high), to constrain the models to the respective predicted patterns. The implementation of the model can be found at https://osf.io/ek5ya.

Targets Left Behind (TLB): The model structure and priors for the TLB models are listed below. All priors were chosen to cover large ranges of plausible values and, importantly, not give advantage to any condition. Variable $y_{i,j}$ refers to the TLBs observed for each participant i in each condition j.

Common part

$$\begin{aligned}
\log n_j^* &\leftarrow \log(N) \\
\log \eta_j &\sim \text{Logistic}(\log n^*, 1) \\
\eta_j &\leftarrow \exp(\log \eta_j) \\
\alpha_j &\leftarrow p_j \cdot \eta_j \\
\beta_j &\leftarrow (1 - p_j) \cdot \eta_j \\
p_{i,j} &\sim \text{Beta}(p_j, \alpha_j, \beta_j) \\
y_{i,j} &\sim \text{Binomial}(p_{i,j}, N)
\end{aligned}$$

Parameter estimation:

$$p^{\text{mean}}_{j\in\{\text{rel,sim,nont,opp}\}} \sim \text{Beta}(1, 1)$$

Model comparison (Relational):

$$\begin{aligned}
p_{\text{midpoint}} &\sim \text{Normal}(0, 1.5) \\
\Delta p_{\text{offset}} &\sim \text{HalfNormal}(\sigma = 0.5) \\
p_{\text{high}(j\in\{\text{rel,sim}\})} &\leftarrow \text{invlogit}(p_{\text{midpoint}} + \Delta p_{\text{offset}}) \\
p_{\text{low}(j\in\{\text{nont,opp}\})} &\leftarrow \text{invlogit}(p_{\text{midpoint}} - \Delta p_{\text{offset}})
\end{aligned}$$

Model comparison (Feature-specific):

$$\begin{aligned}
p_{\text{medium}(j\in\{\text{rel,nont}\})} &\sim \text{Normal}(0, 1.5) \\
\Delta p_{\text{high}} &\sim \text{HalfNormal}(\sigma = 0.5) \\
\Delta p_{\text{low}} &\sim \text{HalfNormal}(\sigma = 0.5) \\
p_{\text{high}(j\in\{\text{sim}\})} &\leftarrow \text{invlogit}(p_{\text{medium}} + \Delta p_{\text{high}}) \\
p_{\text{low}(j\in\{\text{opp}\})} &\leftarrow \text{invlogit}(p_{\text{medium}} - \Delta p_{\text{low}})
\end{aligned}$$

The general rationale of this model follows the beta-binomial model described by Albert and Hu (2019; see [3], pp. 381–385). The different versions for parameter estimation and model comparison follow the same logic as described above for the RoR. The implementation of the model can be found at https://osf.io/43xk6.

Visualisations of the prior

Rate of return (RoR): Supplementary Figure S2. Shows visualisations of the priors on the group level. As can be seen, a wide range of RoRs is covered by the prior distributions, from values close to zero to values larger than two. Since the stimuli were moving, a rate of two items per second already reflects a high rate of return. Wolfe et al.[4], for instance, found click rates between 0.5 and 1 items/s in their foraging tasks with moving stimuli. The posteriors for the RoR obtained in the present study varied across the different conditions between about 1 to 1.2 items/s (see Figure 4 in the main text). Moreover, these parameters concern the central tendency in the group, and individuals could still deviate substantially from these means. Importantly, in the free-parameter model (Fig. S2a), all four conditions receive the same priors, whereas the priors for relational (Fig. S2a) and the feature-specific versions (Fig. S2b) reflect the rank order of the conditions imposed by these models. This results in small differences in the means that reflect the order (see white points inside the violins). Nevertheless, the priors span over wide ranges (Supplementary Fig. S3 shows exemplary draws that further visualise the relationships between the conditions). Taken together, this indicates that the prior was sufficiently vague so as not to distort the posteriors.

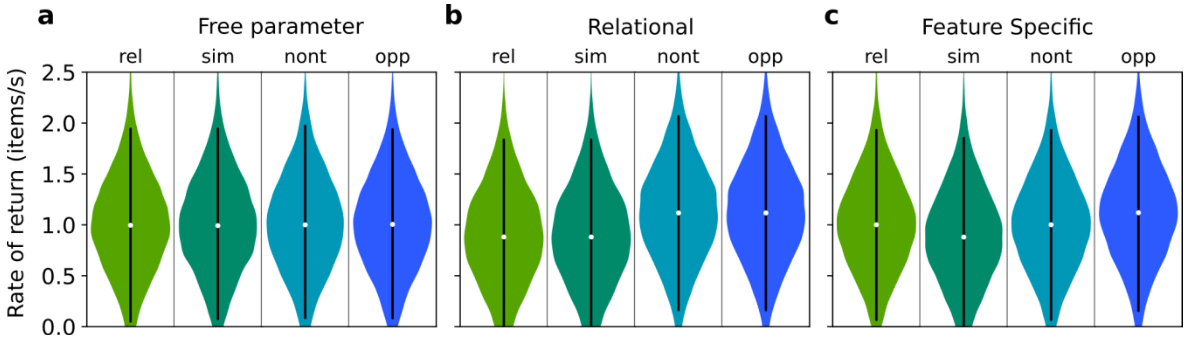

*Supplementary Figure S2. Visualisation of the prior for the central tendencies in the four conditions (rel: Relational, sim: Similar, nont: Non-target, opp: Opposite), for (**a**) the free-parameter model used in parameter estimation and the (**b**) relational and (**c**) feature-specific versions used in the model comparisons.*

The lines in Figure S3 connect the points of values from the same draw. As can be seen, the combinations reside at various y-axis levels, from minimal values up to roughly two items per second. The ordering imposed by these model versions can result in differences of very variable magnitudes (whereas the direction is fixed). For instance, one of the exemplary draws in Fig. S3a (lower one of the two orange lines) results in a difference (between [rel, sim] and [nont, opp]) of more than 0.5 items/s, whereas the draw represented in the olive line (at a similar level as the orange one) is almost entirely flat with only a very small difference between the conditions. (Note that the prior can include values slightly below zero, as the means are additive combinations of unbounded normal distributions; such values are overruled when the posterior is sampled, as there are no negative data points.) In Fig. S3b, similar examples can be found but for the pattern associated with the feature-specific model. Given that the differences in the posterior (Figure 4a in the main text) are about 0.15 items/s, the prior (allowing substantially larger and smaller differences) does not restrict the parameters too much, not distorting the outcomes.

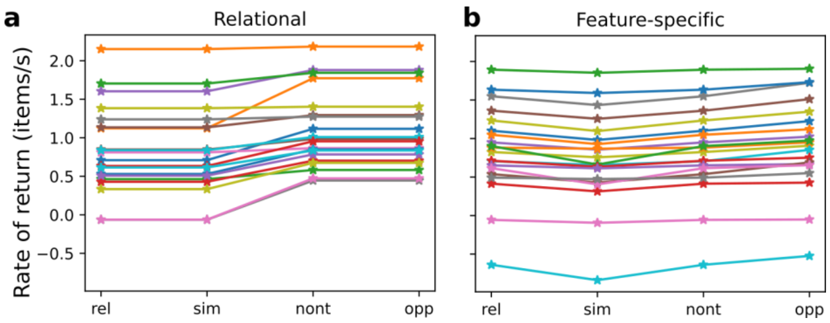

*Supplementary Figure S3. Combination of the effective group-level means drawn from the prior in the four conditions (rel: Relational, sim: Similar, nont: Non-target, opp: Opposite), for (**a**) the relational and (**b**) feature-specific model versions used in the model comparisons.*

Targets left behind (TLB): Supplementary Figures S4 and S5 below show an assessment of prior distributions for the "targets left behind" variable performed in the same way as above for the rate of return and with the same implications: Distributions and differences are sufficiently vague (e.g., the a priori probabilities are high for proportions anywhere between 0 and 1 in the free-parameter model). Note that the somewhat triangular distribution shapes for the relational and feature-specific model versions result from order constraints and the fact that the variable is bounded in the 0 to 1 range.

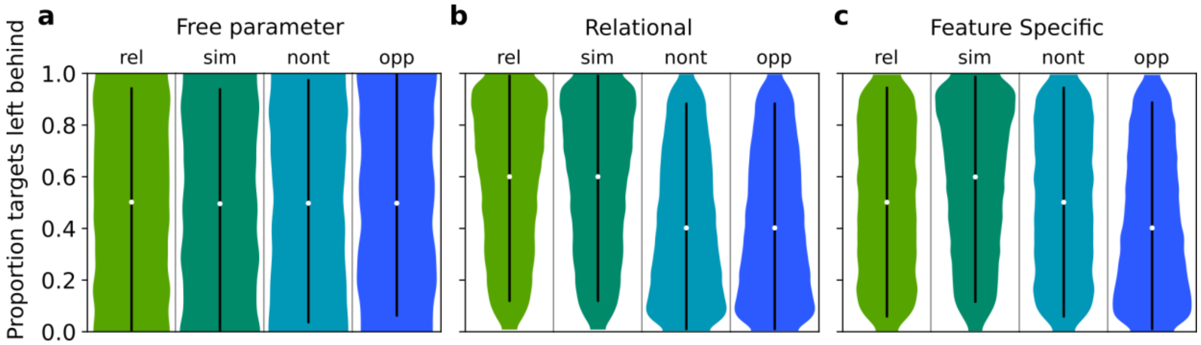

*Supplementary Figure S4. Visualisation of the prior for the central tendencies in the four conditions (rel: Relational, sim: Similar, nont: Non-target, opp: Opposite), for (**a**) the free-parameter model used in parameter estimation and the (**b**) relational and (**c**) feature-specific versions used in the model comparisons.*

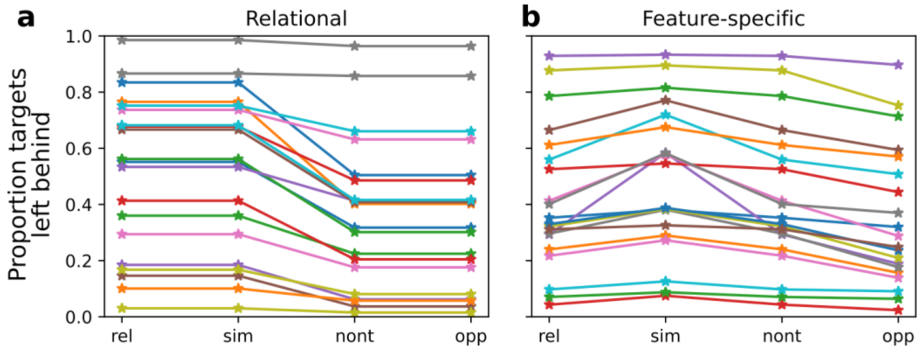

*Supplementary Figure S5. Combination of the effective group-level central tendencies drawn from the prior in the four conditions (rel: Relational, sim: Similar, nont: Non-target, opp: Opposite), for (**a**) the relational and (**b**) feature-specific model versions used in the model comparisons.*

References for the Supplementary Information

1. Tünnermann, J., Chelazzi, L. & Schubö, A. How feature context alters attentional template switching. *J. Exp. Psychol. Hum. Percept. Perform.* **47,** 1431–1444, DOI: https://doi.org/10.1037/xhp0000951 (2021).
2. Vehtari, A., Simpson, D., Gelman, A., Yao, Y., & Gabry, J. Pareto smoothed importance sampling. Preprint at https://arxiv.org/abs/1507.02646 (2022).
3. Albert, J. & Hu, J. *Probability and Bayesian Modeling*. (Chapman and Hall/CRC) DOI: https://doi.org/10.1201/9781351030144 (2019).
4. Wolfe, J. M., Cain, M. S., & Alaoui-Soce, A. Hybrid value foraging: How the value of targets shapes human foraging behavior. *Atten. Percept. Psychophys*, **80**, 609–621. DOI: https://doi.org/10.3758/s13414-017-1471-x (2018).
